# Identification of the metabolic state of surviving cardiomyocytes in the human infarcted heart by spatial single-cell transcriptomics

**DOI:** 10.1097/CP9.0000000000000038

**Published:** 2023-04-04

**Authors:** Yan Shen, Il-man Kim, Neal L. Weintraub, Yaoliang Tang

**Affiliations:** 1 Medical College of Georgia, Augusta University, Augusta, GA 30912, USA.; 2 Anatomy, Cell Biology & Physiology, School of Medicine, Indiana University, Indianapolis, IN 46202, USA.

**Keywords:** Cardiac myocytes, Metabolism, Myocardial infarction, Single-cell RNA-Seq

## Abstract

The metabolic status of surviving cardiomyocytes (CM) in the myocardial tissues of patients who sustained myocardial infarction (MI) is largely unknown. Spatial single-cell RNA-sequencing (scRNA-seq) is a novel tool that enables the unbiased analysis of RNA signatures within intact tissues. We employed this tool to assess the metabolic profiles of surviving CM in the myocardial tissues of patients post-MI.

**Methods:**

A spatial scRNA-seq dataset was used to compare the genetic profiles of CM from patients with MI and control patients; we analyzed the metabolic adaptations of surviving CM within the ischemic niche. A standard pipeline in Seurat was used for data analysis, including normalization, feature selection, and identification of highly variable genes using principal component analysis (PCA). Harmony was used to remove batch effects and integrate the CM samples based on annotations. Uniform manifold approximation and projection (UMAP) was used for dimensional reduction. The Seurat “FindMarkers” function was used to identify differentially expressed genes (DEGs), which were analyzed by the Gene Ontology (GO) enrichment pathway. Finally, the scMetabolism R tool pipeline with parameters method = VISION (Vision is a flexible system that utilizes a high-throughput pipeline and an interactive web-based report to annotate and explore scRNA-seq datasets in a dynamic manner) and metabolism.type = Kyoto Encyclopedia of Genes and Genomes (KEGG) was used to quantify the metabolic activity of each CM.

**Results:**

Analysis of spatial scRNA-seq data showed fewer surviving CM in infarcted hearts than in control hearts. GO analysis revealed repressed pathways in oxidative phosphorylation, cardiac cell development, and activated pathways in response to stimuli and macromolecular metabolic processes. Metabolic analysis showed downregulated energy and amino acid pathways and increased purine, pyrimidine, and one-carbon pool by folate pathways in surviving CM.

**Conclusions:**

Surviving CM within the infarcted myocardium exhibited metabolic adaptations, as evidenced by the downregulation of most pathways linked to oxidative phosphorylation, glucose, fatty acid, and amino acid metabolism. In contrast, pathways linked to purine and pyrimidine metabolism, fatty acid biosynthesis, and one-carbon metabolism were upregulated in surviving CM. These novel findings have implications for the development of effective strategies to improve the survival of hibernating CM within the infarcted heart.

## INTRODUCTION

Following myocardial infarction (MI), myocardial tissue contains a mixture of dead and viable cardiomyocytes (CM), the balance of which depends on factors such as the area at risk and duration of ischemia^[1]^. Little is known about how surviving CM flexibly reprogram their metabolic energy within infarcted heart tissues. Xu *et al.*^[2]^. recently reported that the metabolic energy of ischemic CM switches from mitochondrial oxidative phosphorylation (OXPHOS) to glycolysis and lactic fermentation. Moreover, upregulated Glucose transporter type 4 (GLUT4) expression and increased glucose uptake in the ischemic myocardium were detected by Lu *et al.*, suggesting a protective response to preserve the viability of ischemic CM^[3]^. However, technical limitations have prevented the assessment of CM metabolism in infarcted heart tissue.

Single-cell RNA-Seq (scRNA-seq) is a revolutionary technique that allows the unbiased identification of gene expression heterogeneity at the cellular level. Recently, spatial scRNA-seq has been employed to define the gene expression profiles of cells within tissues, allowing unparalleled resolution of tissue gene expression in disease states. Exploiting this methodology to assess gene expression in surviving CM within the infarcted niche could advance the understanding of how vulnerable CM adjust their metabolism to survive in this microenvironment.

Altered gene expression and metabolic activity in surviving CM in infarcted hearts compared to control hearts may contribute to functional impairment following myocardial infarction (MI). The major purpose of this study was to identify these metabolic changes, which may enable the development of therapeutic strategies to promote CM survival and functional recovery after MI. We compared the gene expression in control and infarcted human heart CM using spatial scRNA-seq. Pathways such as oxidative phosphorylation and cardiac cell development were inhibited, while responses to stimuli and macromolecular metabolic processes were activated. Metabolic activity was reduced in oxidative phosphorylation, citrate cycle, glycolysis/gluconeogenesis, pyruvate metabolism, and pentose phosphate pathway, and “fatty acid degradation” was inhibited. In amino acid metabolism, metabolic activities were reduced in alanine, aspartate, glutamate, arginine, proline, cysteine, methionine, glutathione, tyrosine, and tryptophan. Purine and pyrimidine metabolism were also upregulated. These findings may improve therapeutic strategies for increasing CM survival and recovery rate after MI.

## METHODS

### Spatial single-cell RNA-sequencing datasets

The processed spatial scRNA-seq datasets were downloaded from Zenodo (https://zenodo.org/record, DOI:10.5281/zenodo.6578047)^[4]^. To minimize the effects of age and sex on data analysis, only spatial scRNA-seq transcriptome datasets of control_P1 (normal heart of a 44-year-old male patient, 11,416 cells in total) and IZ_BZ-P2 [heart of a 44-year-old male patient 5 days after acute MI, ischemic zone (IZ), and border zone (BZ), 1,897 cells in total] were selected for this study (Table 1).

**Table 1:** Heart samples for spatial transcriptomic analysis

Patient_region_ID	Control patient #1 (P1)	Ischemia zone/border zone of patient #2 (IZ/BZ_P2)
Region_zone_description	Control	IZ
Age (years)	44	44
Gender	Male	Male
Infarction location	n.a.	Anterior left ventricular due to occlusion of the left coronary artery
Days after infarction	Control	5
Disease	Control	Acute myocardial infarction
Pathologist’s description; HE staining cryo section	Normal; Myocardium	Ischemic left side slide normal BZ; Necrotic CM, edema, neutrophils

CM: cardiomyocytes; IZ: ischemic zone; BZ: border zone; HE: hematoxylin and eosin; ID: identity; n.a.: not available.

### ScRNA-seq data processing, cell-type annotation, quality control, and data visualization

The Seurat R package (V4.2) was used for downstream analytical procedures: cells with extreme feature counts (<200 or >3,000) and >50% reads with mitochondrial alignment were removed. Subsequently, we performed data normalization, high-variance feature identification, data scaling, and principal component analysis (PCA) using Seurat’s classic workflow. Then, the Harmony algorithm (V0.1) was used to correct the potential batches between CM samples and to integrate them according to the annotations. Next, dimensional reduction was performed using the Uniform Manifold Approximation and Projection (UMAP) with the parameter “reduction” set as “harmony.” Seurat’s “FindNeighbors” and “FindClusters” functions were applied to cell clustering analysis. CM were annotated according to the expression of typical cellular markers, such as Ryanodine receptor 2 (RYR2), Myosin Heavy Chain 7 (MYH7), and Titin (TTN), and only CM clusters were retained. “UMAP” was used to visualize the CM, of which there were four distinct clusters (0–3), under control and MI conditions.

### Differential expression analysis and pathway enrichment analysis

Differentially expressed genes (DEGs) between CM from infarcted hearts (both infarct and BZ) and control hearts were identified using the Seurat “FindMarkers” function under the default Wilcoxon rank-sum test. The *P* values <0.05 and estimated log2-fold changes >2 were considered significantly differentially abundant.

We applied the gseGO function from R/Bioconductor “clusterProfiler package” (V4.4.4) and org.Hs.eg.db (V3.15) to perform gene ontology (GO) and pathway enrichment analyses of DEGs with default parameters. Statistical significance was set at *P* < 0.05.

### Single-cell metabolic analysis

To discern the metabolic adaptations of CM from infarcted hearts in spatial scRNA-seq datasets, we applied the “scMetabolism” package to quantify the metabolic activities of every CM in control and infarcted hearts. Specifically, the method was set to “VISION,” and analyzed using the Kyoto Encyclopedia of Genes and Genomes metabolic gene sets.

### Statistical analysis

All statistical analyses and data presentation were performed using R program version 4.2.0. For all scMetabolism score comparisons, statistical analyses were completed using GraphPad Prism version 9.41 (GraphPad Software, LLC.USA) *t* tests unless otherwise indicated, and *P*<0.05 was considered statistically significant. GO bioprocess (GO-BP) and Gene Set Enrichment Analysis (GSEA) analysis was performed by using R package clusterProfiler version 3.8.1, R package DOSE (version 3.6.1), and R package org.Hs.eg.db version 3.6.0. The analysis results were visualized by using the R package Enrichplot version 1.2.0.

## RESULTS

### Identification of CM clusters in infarcted hearts by spatial scRNA-seq analysis

**Figure 1.**
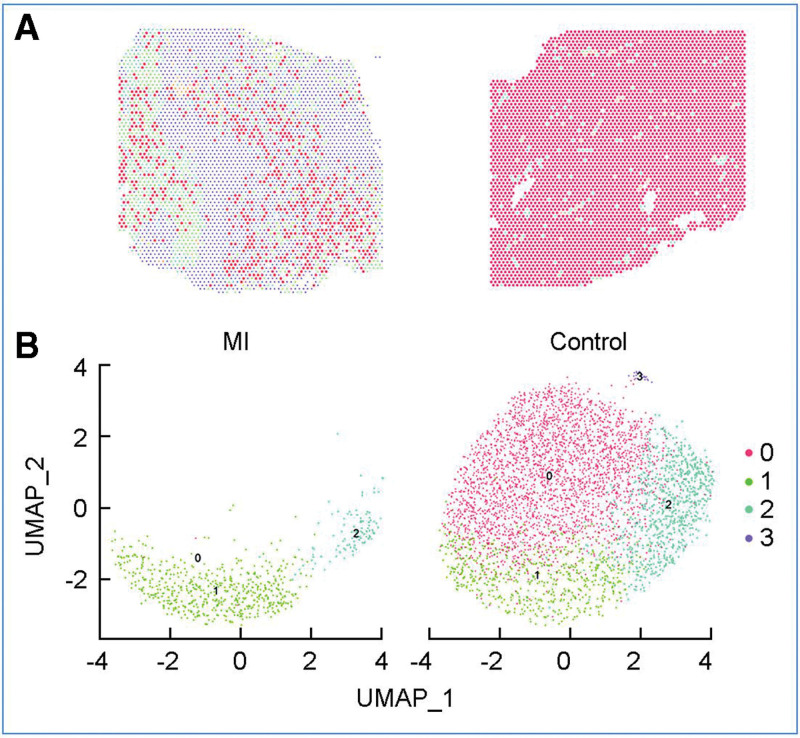
**Spatial transcriptomics data from human control and MI hearts. A**, Spatial patterns found in the human hearts [red dot: cardiomyocytes (CM)]. The left panel shows the infarcted heart section, and the right panel illustrates the control heart. **B**, CM subpopulations in the infarcted heart (left) and control heart (right) for each cell population on the two-dimensional Uniform Manifold Approximation and Projection (UMAP) map. UMAP_1 and UMAP_2 correspond to the first and second dimensions, respectively, of the reduced-dimensional graph projection. CM: cardiomyocytes; MI: myocardial infarction.

**Figure 2.**
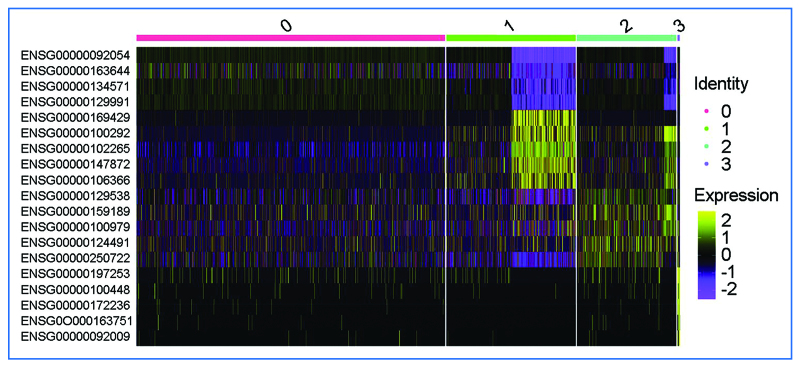
**Heatmaps depicting the top five genetic markers used to classify CM subpopulations in the human hearts and their expression levels.** CM: cardiomyocytes.

We performed an unbiased pathway enrichment analysis of published spatial snRNA-seq data from human hearts with or without MI, selecting single-cell transcriptome profiles generated from heart samples of control male patients and patients with MI of the same age. After a rigorous quality control procedure, we obtained a sparse matrix containing 4,029 CM in control hearts and 666 CM in infarcted hearts. Potential batch effects between samples processed in different batches were eliminated using the RunHarmony function in the Harmony package, before performing unsupervised graph-based clustering analysis. Subsequently, we segmented CM based on the expression of typical CM markers (RYR2, MYH7, and TTN) and integrated cells using the Harmony package for downstream analysis. We performed unbiased clustering of the transcriptional profiles of CM in each sample (clusters 0, 1, 2, and 3) (Figure 1). The clustering results showed that CM from control hearts was predominantly contained in cluster 0. Notably, the surviving CM in infarcted hearts was almost exclusively contained in clusters 1 and 2. Heatmaps depicting the relative expression levels of genetic markers used to classify the CM subpopulations are shown in Figure 2. These results suggest that surviving CM in infarcted heart tissue exhibit a phenotypic shift, with clusters 1 and 2 potentially representing subgroups that are more resistant to ischemic injury.

### Differential gene expression profiling of CM in control versus infarcted hearts

**Figure 3.**
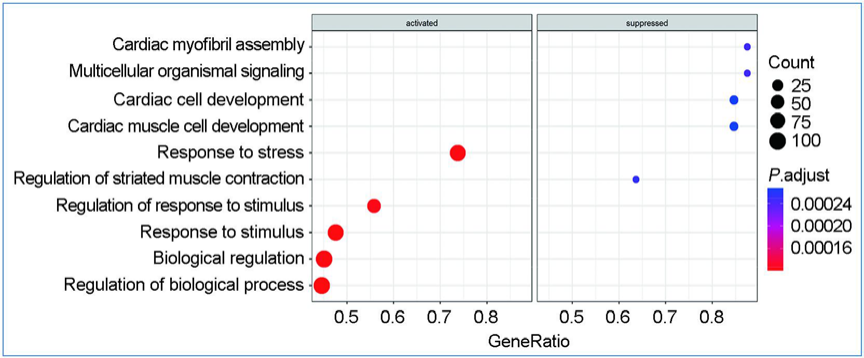
**Significantly enriched (activated and suppressed) GO-BP pathways in surviving CM from infarcted hearts.** The vertical terms are the names of GO-BP terms, and the length of horizontal graph represents the gene ratio. The area of circle in the graph indicates the gene counts. The depth of the color represents the adjusted *P*-value. CM: cardiomyocytes; GO-BP: Gene Ontology bioprocess.

**Figure 4.**
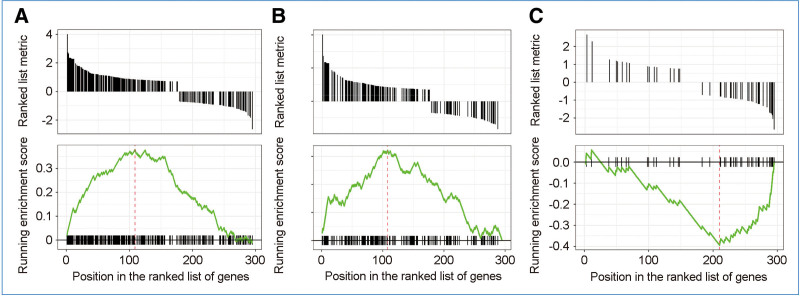
**GO-BP-enrichment plots of representative gene sets for GSEA-based pathways. A**, Response to stimulus; **B**, Negative regulation of metabolic process; **C**, Striated muscle contraction. GO-BP: Gene Ontology bioprocess; GSEA: Gene Set Enrichment Analysis.

To investigate the biological processes of differentially expressed genes (DEGs) between CM residing in infarcted and control hearts, we performed GO enrichment analysis using the clusterProfiler package. GO analysis identified that “suppressed oxidative phosphorylation,” “cardiac cell development,” and “cardiac muscle cell development” was significantly inhibited in CM within infarcted tissues (Figure 3). Conversely, the GO terms “response to stimulus,” “positive regulation of biological process,” and “macromolecule metabolic process” were significantly upregulated in these CM, suggesting that CM in the infarcted heart experience stress conditions in which oxidative phosphorylation is inhibited. In addition, Gene set enrichment analysis (GSEA) revealed that the gene sets representing pathways of “response to stimulus” and “negative regulation of metabolic process” were significantly upregulated (Figure 4A and 4B), but those representing the “striated muscle contraction” were downregulated in CM residing in infarcted heart tissue (Figure 4C).

### Differential metabolic pathways of cardiomyocytes within infarcted and the control hearts

Myocardial ischemia and reperfusion (I/R) lead to an inflammatory response that causes cardiac injury and remodeling. Several pro-inflammatory cytokines, including tumor necrosis factor-α (TNF-α), interleukin-1α, interleukin-6, macrophage migration inhibitory factor, and D-dopamine isotopes, have been shown to improve cell survival and compensate for energy deprivation during I/R^[5]^. To investigate whether surviving CM in infarcted heart tissue exhibit metabolic adaptations, we investigated the metabolic landscape using the “scMetabolism” package, which focuses on a comprehensive collection of metabolic pathways. First, we filtered the metabolic pathways and compared the activity of CM in infarcted and control hearts. These results suggest a significant difference in the enrichment of multiple metabolic pathways.

**Figure 5.**
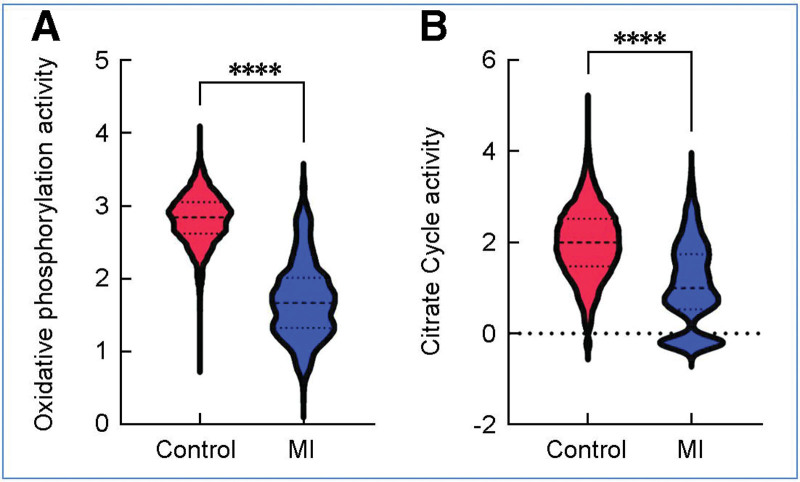
**OXPHOS metabolic pathway activities in CM in control versus infarcted heart tissues. A**, Violin plots comparing the OXPHOS pathway scores. **B**, Violin plots comparing citric acid cycle (TCA cycle) pathway scores. Control: *n* = 4,027, MI: *n* = 664, **** *P* < 0.0001. CM: cardiomyocytes; MI: myocardial infarction; OXPHOS: oxidative phosphorylation.

**Figure 6.**
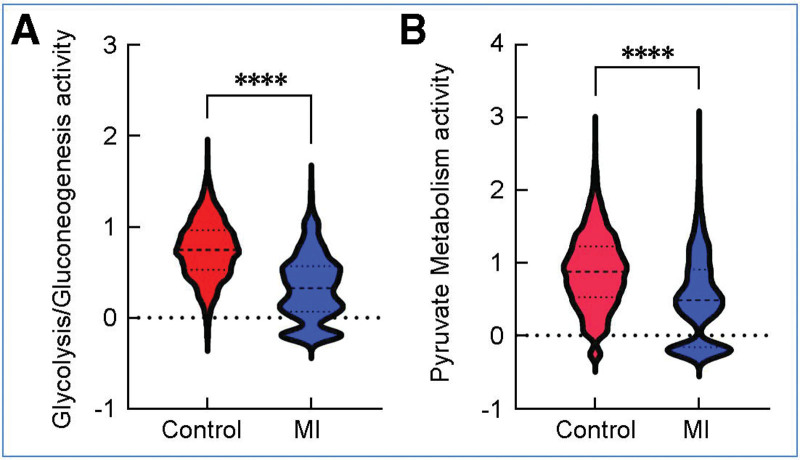
**Glycolysis metabolic pathway activities in CM in control versus infarcted heart tissues. A**, Violin plots comparing glycolysis/gluconeogenesis pathway scores. **B**, Violin plots comparing pyruvate metabolism pathway scores. Control: *n* = 4,027, MI: *n* = 664, **** *P* < 0.0001. CM: cardiomyocytes; MI: myocardial infarction.

**Figure 7.**
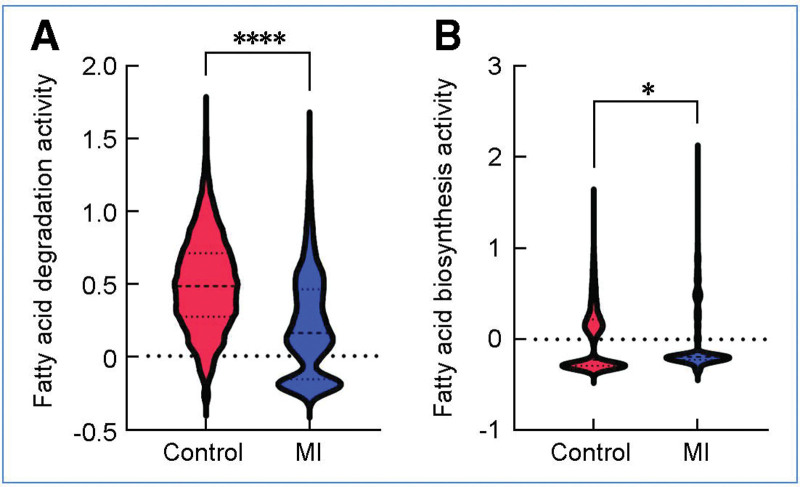
**Lipid metabolic pathway activities in CM in control and infarcted heart tissues. A**, Violin plots comparing the fatty acid degradation. **B**, Violin plots comparing fatty acid biosynthesis pathway scores. Control: *n* = 4,027, MI: *n* = 664, *P < 0.05, ****P < 0.0001. CM: cardiomyocytes; MI: myocardial infarction.

**Figure 8.**
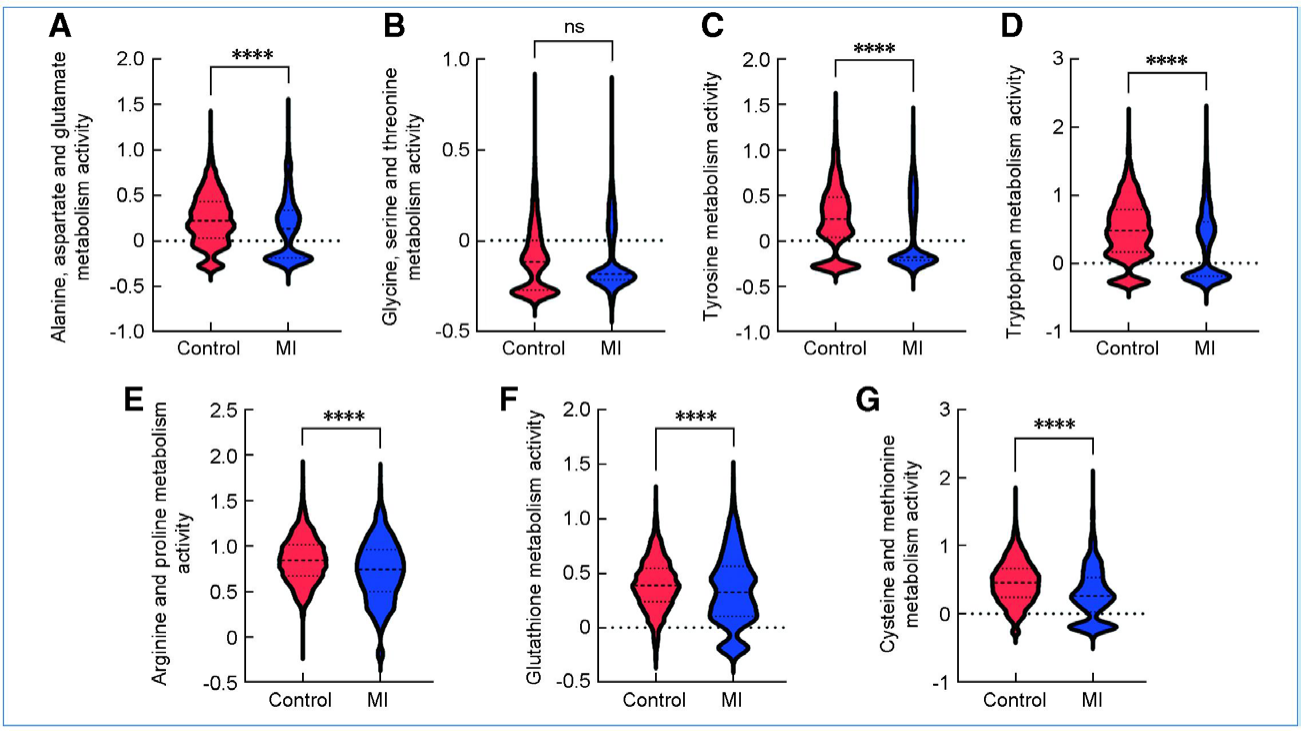
**Amino acid metabolic pathway activities in CM in control and infarcted heart tissues. A**, Violin plots comparing alanine, aspartate, and glutamate metabolism pathway scores. **B**, Violin plots comparing glycine, serine, and threonine metabolism pathway scores. **C**, Violin plots comparing tyrosine metabolism pathway scores. **D**, Violin plots comparing tryptophan metabolism pathway scores. **E**, Violin plots comparing arginine and proline metabolism pathway scores. **F**, Violin plots comparing glutathione metabolism pathway scores. **G**, Violin plots comparing cysteine and methionine metabolism pathway scores. Control: *n* = 4,027, MI: *n* = 664, ****P < 0.0001. CM: cardiomyocytes; MI: myocardial infarction; ns: no significant difference.

**Figure 9.**
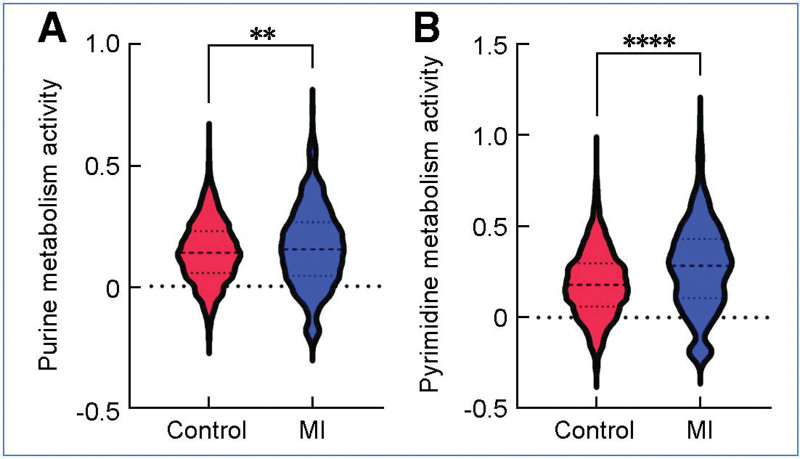
**Nucleic acid metabolic pathway activities in CM in control and infarcted heart tissues. A**, Violin plots comparing purine metabolism pathway scores. **B**, Violin plots comparing pyrimidine metabolism pathway scores. Control: *n* = 4,027, MI: *n* = 664, **P < 0.01, ****P < 0.0001. CM: cardiomyocytes; MI: myocardial infarction.

**Figure 10.**
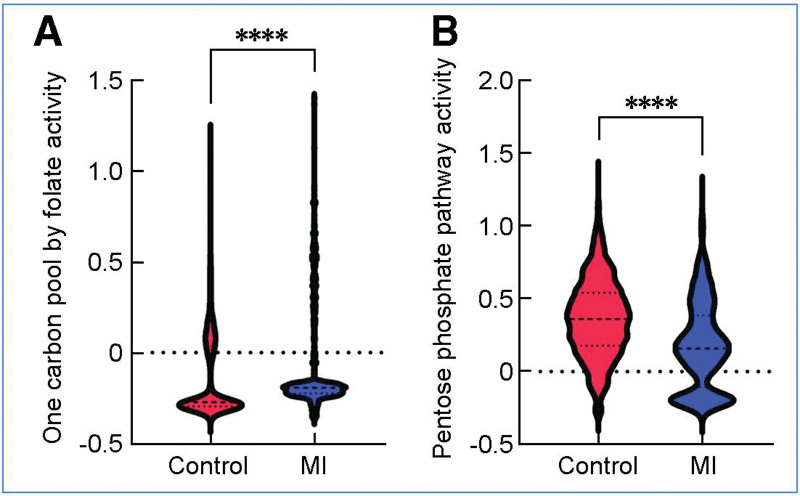
**One-carbon metabolism-related metabolic pathway activities in CM from control and infarcted heart tissues. A**, Violin plots comparing one-carbon pool by folate pathway scores. **B**, Violin plots comparing pentose phosphate pathway scores. Control: *n* = 4,027, MI: *n* = 664, ****P < 0.0001. CM: cardiomyocytes; MI: myocardial infarction.

1. OXPHOS and the citric acid cycle (TCA cycle) are the major energy pathways associated with mitochondria^[6,7]^. ScMetabolism analysis indicated reduced activities of OXPHOS and the TCA cycle in CM from infarcted hearts than with control hearts (Figure 5A and 5B).
2. Ischemic tissues are highly dependent on glycolysis and pyruvate metabolism for adenosine triphosphate (ATP) production. Pyruvate metabolism is a key step in glycolysis/gluconeogenesis. Both “glycolysis/gluconeogenesis” and “pyruvate metabolism” activities were significantly reduced in CM in infarcted hearts than in control hearts (Figure 6A and 6B).
3. Under controlled conditions, the heart balances fatty acid uptake, metabolism, and oxidation to maintain energy generation, membrane biosynthesis, and lipid signaling^[8]^. Compared with CM in control hearts, “the fatty acid degradation” was significantly reduced in CM from infarcted hearts (Figure 7A), while the activity of “fatty acid biosynthesis” was significantly increased (Figure 7B).
4. Amino acids are used as metabolic substrates in the ischemic heart^[9]^, and also as diagnostic and prognostic markers for heart failure^[10]^. Our analysis suggests that multiple amino acid metabolic pathways, including “alanine, aspartate, glutamate, cysteine, methionine, glutathione, arginine, tyrosine, and tryptophan metabolism,” were significantly downregulated in CM from infarcted hearts when compared to control hearts. However, “glycine, serine, and threonine metabolism” was not significantly different between the two groups (Figure 8A–8G).
5. Purine and pyrimidine metabolism are crucial for nucleotide and nucleoside synthesis. In heart failure, the intracellular concentration of total adenine nucleotides decreases, and ATP is replenished through salvage and/or de novo synthesis pathways using purine nucleotide metabolism^[11]^. Our data suggest that compared to CM in control hearts, CM in infarcted hearts exhibited increased purine and pyrimidine metabolic activity (Figure 9A and 9B).
6. Serine- and glycine-derived one-carbon metabolism is used in the folate cycle, which contributes to cardio-protection by generating reduced nicotinamide adenine dinucleotide phosphate (NADPH), nicotinamide adenine dinucleotide (NADH), and ATP in response to oxidative stress. The folate cycle is also essential for nucleotide and glutathione synthesis^[12–14]^. Our data identify increased “one carbon pool by folate” activity in CM from infarcted versus control hearts (Figure 10A). The pentose phosphate pathway contributes to reducing equivalents required to regenerate NADPH-dependent antioxidant glutathione, which directly and indirectly protects against oxidative stress^[12,15]^. Interestingly, our data suggest reduced pentose phosphate activity in CM from infarcted hearts compared to that in CM from control hearts (Figure 10B).

## DISCUSSION

In this study, we analyzed spatial single-cell transcriptomics data to assess metabolic adaptations of CM surviving in heart tissue post-MI. Our analysis suggests that surviving CM within the ischemic niche exhibits specific signatures of metabolic reprogramming of multiple pathways, including OXPHOS, glycolysis, the pentose phosphate pathway, amino acid and nucleic acid metabolism, and fatty acid biosynthesis. These data may provide new insights into the metabolic fate of surviving CM after MI. Moreover, a better understanding of the metabolic adaptations of surviving CM post-MI might can lead to novel therapeutic strategies for patients with ischemic cardiomyopathy.

Mitochondrial OXPHOS yields as a byproduct reactive oxygen species (ROS), which in turn can damage mitochondrial DNA, impair respiratory chain function, and lead to nuclear DNA damage^[16,17]^. Not only are CM able to combat the effects of ROS produced during normal heart function they are also capable of metabolically adapting to ensure viability under conditions of stress as well as, in pathological states such as ischemia. To identify the unique metabolic adaptations of surviving CM in infarcted hearts, we performed spatial scRNA-seq data analyses and compared them with datasets from age- and sex-matched control hearts. Our GO-BP analysis suggests that surviving CM in infarcted hearts has reduced OXPHOS and TCA cycle activities, which may be beneficial for protecting ischemic CM from ROS damage. For example, short-term serine/threonine-protein kinase (AKT) activation has been reported to exert cardioprotective effects by reducing OXPHOS^[18,19]^. Oxygen and nutrient deprivation associated with ischemia and acidification in infarcted heart tissues may be key factors in the inhibition of the citric acid cycle (TCA cycle) and OXPHOS, and inhibition of mitochondrial energy production may in turn facilitate CM survival in this microenvironment^[20]^.

Fatty acids are essential for normal heart function, serving as preferred energy substrates through efficient ATP production and replenishing lipid pools in the cell membranes^[21]^. In this study, we found that surviving CM in infarcted hearts exhibited evidence of dysregulation of fatty acid metabolic pathways, including the downregulation of fatty acid degradation and upregulation of fatty acid biosynthesis. Shekar *et al.*^[22]^. found that increased degradation of key proteins involved in mitochondrial fatty acid metabolism resulted in impaired fatty acid oxidation in heart failure. The increased fatty acid biosynthesis detected in surviving CM in infarcted heart tissues may promote membrane repair and could be particularly important for restoring damaged mitochondria^[23]^.

The metabolism of CM in ischemic heart tissues is marked by a switch from mitochondrial fatty acid oxidation to glycolysis, which contributes to ATP production essential for maintaining CM survival under these conditions^[2,24–26]^. Surprisingly, scMetabolism analysis suggested that surviving CM suppressed glycolysis/gluconeogenesis and pyruvate metabolism. The mechanisms by which glycolysis might be inhibited in surviving CM remain unclear. Sustained ischemia may lead to intracellular glucose deprivation, resulting in the inhibition of glycolysis^[27]^. Whether this might be pathological or protective is unclear. Recent studies have reported that inhibition of glycolysis with 2-Deoxy-D-glucose (2-DG), a glycolysis inhibitor, attenuates cardiac fibrosis after MI^[28]^.

In a normally functioning heart, amino acids do not play a major role as energy substrates. ScMetabolism analysis revealed that surviving CM exhibited reduced activity in several amino acid metabolic pathways, including metabolism of alanine, aspartate, glutamate, cysteine, methionine, glutathione, arginine, proline, tyrosine, and tryptophan. Pisarenko *et al*^[29]^. reported that ischemia/reperfusion injury results in a significant decrease in total glutamate and aspartate pools in isolated guinea pig hearts. Cysteine and methionine metabolism is important for the synthesis of antioxidants (e.g., glutathione) and methyl donors (S-adenosylmethionine)^[30]^. Downregulation of cysteine and methionine metabolism, as well as glutathione metabolism, may impair redox homeostasis and render CM vulnerable to ROS-induced injury. Tyrosine and tryptophan are used to produce hormones or neurotransmitters such as epinephrine and noradrenaline^[31]^. Downregulation of arginine and proline metabolism has been reported to be involved in the progression of heart failure^[32]^, and targeting arginine and proline metabolism has been suggested as a potential strategy for the treatment of heart failure^[33]^.

Nucleotide metabolism, including purine and pyrimidine metabolism, is an alternative metabolic pathway that avoids the effects of glucose starvation^[34]^. ScMetabolism analysis suggests that surviving CMs exhibit upregulated purine and pyrimidine metabolism, which may be beneficial in protecting ischemic CM from glucose starvation in the ischemic environment. In addition, the increased activity of pyrimidine and purine metabolism implies an increased demand for nucleotides, which is a typical feature of proliferating cells, and for DNA repair and biosynthesis owing to oxidative stress-induced DNA damage^[35,36]^.

One-carbon metabolism is essential for a variety of physiological processes, including nucleotide metabolism, glutathione (synthesized from glutamate, cysteine, and glycine), and NAPDH synthesis for antioxidant purposes^[37]^. One-carbon pools regulate the conversion of serine to glycine in the mitochondria and release the folate produced into the cytosol for purine synthesis^[38]^. One-carbon pools also help limit the production of ROS by increasing the production of NADPH in mitochondria^[39]^. In infarcted hearts, surviving CM exhibits evidence of upregulation of the one-carbon pool by folate activity, which would likely promote cell viability in this hostile milieu.

In summary, we present unbiased evidence regarding the metabolic adaptations of individual CM residing in infarcted heart tissues of patients with MI. These findings not only advance our understanding of how CM regulate their metabolic status to survive in a detrimental microenvironment, but also open new possibilities for effectively treating MI patients by targeting key metabolic pathways.

### Limitations

This study has few limitations which are stated as follows:

1. Spatial transcriptomic analysis techniques lack single-cell resolution; combining spatial scRNA-seq with single-cell RNA-seq datasets could help overcome this limitation to allow single-cell resolution^[40,41]^.
2. In addition, data were derived from age- and sex-matched humans with and without MI. We acknowledge that aside from the presence or absence of MI, differences in the patients studied, such as underlying medical conditions, treatments rendered, and factors associated with tissue procurement, could have impacted the results. Nevertheless, our results provide a foundation for future studies designed to specifically investigate the metabolic adaptations of CM in the setting of MI.
3. Additionally, we excluded apoptotic low-quality cells by excluding cells with a high percentage of reads for mitochondrial genomic transcripts and characteristic counts. However, we cannot distinguish “surviving” from “dying” CM by simply setting a threshold for the percentage of mitochondria.
4. Finally, validation is often required for spatial scRNA-seq analyses. We did not validate our scRNA-seq results using quantitative RT-PCR because we had difficulty obtaining left ventricular tissue from healthy individuals and patients with acute MI.

## FUNDING

This work was supported by the American Heart Association Transformational Project Award 964856 (to Y.T.); NIH grants 2R01HL086555 (to Y.T.) and R0HL1134354 (to Y.T./M.A./N.L.W.).

## AUTHOR CONTRIBUTIONS

YS, YT: participated in research design; YT: participated in the data analysis; YT, YS, IK, NLW: participated in the writing of the paper.

## CONFLICTS OF INTEREST STATEMENT

The authors declare that they have no financial conflict of interest with regard to the content of this manuscript.

## DATA SHARING STATEMENT

All data used in this study are publicly available, with detailed access links described in the Methods section. No new algorithms were developed for this study.
